# Supplementary figures and images for: Unlike Brief Inhibition of Microglia Proliferation after Spinal Cord Injury, Long-Term Treatment Does Not Improve Motor Recovery
Source: Brain Sci. 2021 Dec 13;11(12):1643. doi: 10.3390/brainsci11121643 (PMC8699766; doi:10.3390/brainsci11121643)

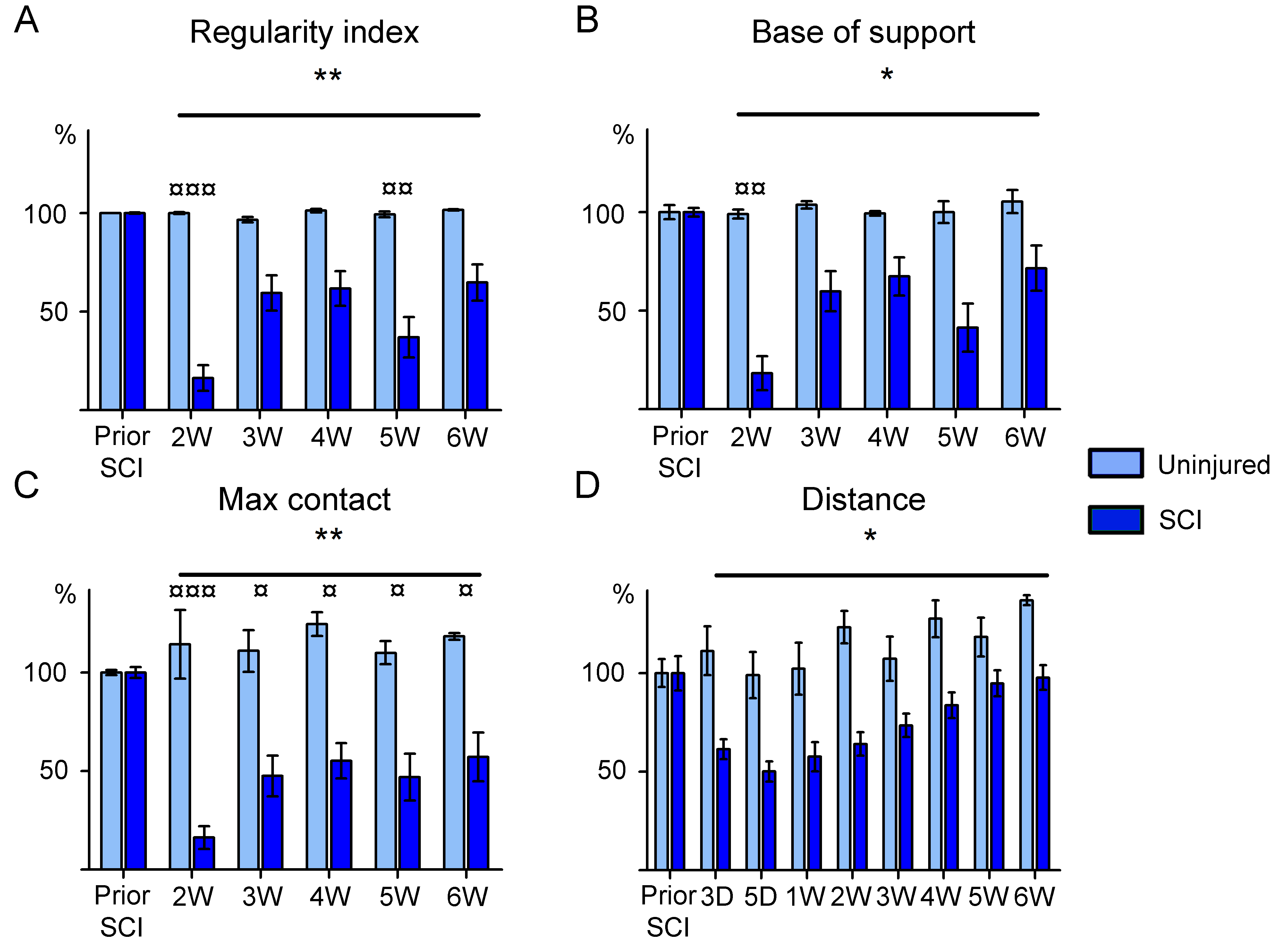

Supplement: Supplementary file 1 [file brainsci-11-01643-s001.zip › brainsci-1463297-supplementary.tif]
